# Identification of V6.51L as a selectivity hotspot in stereoselective A_2B_ adenosine receptor antagonist recognition

**DOI:** 10.1038/s41598-021-93419-x

**Published:** 2021-07-08

**Authors:** Xuesong Wang, Willem Jespers, Rubén Prieto-Díaz, Maria Majellaro, Adriaan P. IJzerman, Gerard J. P. van Westen, Eddy Sotelo, Laura H. Heitman, Hugo Gutiérrez-de-Terán

**Affiliations:** 1 Division of Drug Discovery and Safety, Leiden Academic Centre for Drug Research, Einsteinweg 55, 2333 CC Leiden, The Netherlands; 2 Department of Cell and Molecular Biology and Science for Life Laboratory, Uppsala University, Box 596, 751 24 Biomedical CenterUppsala, Sweden; 3 Centro Singular de Investigación en Química Biolóxica Y Materiais Moleculares (CIQUS), Santiago de Compostela, Spain; 4 Departamento de Química Orgánica, Facultade de Farmacia, Universidade de Santiago de Compostela, 15782 Santiago de Compostela, Spain; 5 Oncode Institute, Leiden, The Netherlands

**Keywords:** G protein-coupled receptors, Computational chemistry, Receptor pharmacology, Lead optimization

## Abstract

The four adenosine receptors (ARs) A_1_AR, A_2A_AR, A_2B_AR_,_ and A_3_AR are G protein-coupled receptors (GPCRs) for which an exceptional amount of experimental and structural data is available. Still, limited success has been achieved in getting new chemical modulators on the market. As such, there is a clear interest in the design of novel selective chemical entities for this family of receptors. In this work, we investigate the selective recognition of ISAM-140, a recently reported A_2B_AR reference antagonist. A combination of semipreparative chiral HPLC, circular dichroism and X-ray crystallography was used to separate and unequivocally assign the configuration of each enantiomer. Subsequently affinity evaluation for both A_2A_ and A_2B_ receptors demonstrate the stereospecific and selective recognition of (*S*)-ISAM140 to the A_2B_AR. The molecular modeling suggested that the structural determinants of this selectivity profile would be residue V250^6.51^ in A_2B_AR, which is a leucine in all other ARs including the closely related A_2A_AR. This was herein confirmed by radioligand binding assays and rigorous free energy perturbation (FEP) calculations performed on the L249V^6.51^ mutant A_2A_AR receptor. Taken together, this study provides further insights in the binding mode of these A_2B_AR antagonists, paving the way for future ligand optimization.

## Introduction

Adenosine receptors (ARs) are a family of G protein-coupled receptors (GPCR) for which an exceptional amount of structural and experimental data is available^1,2^. Still, the number of therapeutic agents on the market that specifically target this family of receptors remains relatively low^3^. On the other hand, selective targeting of any of the four adenosine receptor subtypes (A_1_, A_2A_. A_2B_ and A_3_) provides an interesting avenue to address not only unmet therapeutic needs^4^ and limited off-target effects^5^, but also to help elucidating the (patho)physiological role of the different receptors within the family. One topic that is receiving increasing interest is the molecular mechanisms by which the two A_2_AR subtypes regulate the immune response to tumor growth and metastasis^6^.

**Figure 1:**
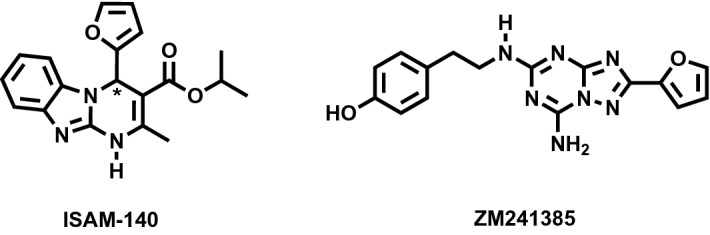
2D representation of the chemical structures of the AR ligands used in this work, i.e. ZM241385, ( ±) ISAM-140, (R)-ISAM-140 and (S)-ISAM-140. The chiral center in ISAM-140 is indicated with an asterisk.

Over the last years, different AR ligands have been developed with optimized selectivity profiles^7–9^. Within these AR ligand design programs, the generation of potent and selective antagonists has allowed the identification of powerful chemical tools to characterize each of the members of this receptor family. Examples include the A_2A_AR selective antagonist ZM241385, and the A_2B_AR selective antagonist ISAM-140, the latter originating from our in-house optimization program (Fig. 1)^7,9–11^. The development of ISAM-140 was done following careful structure-affinity relationship (SAR) modeling, based on a computational binding mode of this chemotype, which suggested an important role of the stereogenic center in the heterocyclic scaffold in its high binding affinity (Fig. 1)^11,12^. The prediction of the active stereoisomer for this chemotype was later confirmed indirectly by experimental characterization of the active stereoisomers for representative compounds of a series of cyanopyrimidines^10^, fluorinated tricyclic derivatives^13^ and aza-bioisosteres of the pentagonal heterocycle^14^. This binding model proposed that the stereospecific complementarity to the A_2B_AR cavity was due to the optimal accommodation of the thiophene/furan ring around the chiral center of the core scaffold (Fig. 1), with the A_2B_AR specific residue V250^6.51^ (Ballesteros Weinstein numbering in superscripts)^15^. Indeed, this valine is replaced by a leucine in all other AR subtypes, which could explain the highly selective profile of these series of non-planar antagonists towards the A_2B_AR.

In this work, we report the chiral separation of ISAM140 and confirm its stereospecific binding mode to the A_2B_AR. An A_2A_AR construct was designed to include the corresponding A_2B_AR valine sidechain (L249V^6.51^ A_2A_AR mutant), which in line with the starting hypothesis partially recovered the affinity for ISAM-140. Interestingly, this effect was observed for both stereoisomers of the antagonist, and is herein explained on the basis of structure-energetic modeling via rigorous free energy perturbation (FEP) calculations. These results validate the proposed role of V250^6.51^ in the A_2B_AR subtype selectivity of these stereospecific chemotype, and paves the road for further design of selective antagonists as well as dual A_2_AR ligands.

## Results

### Generating A_2A_AR-ligand models

**Figure 2:**
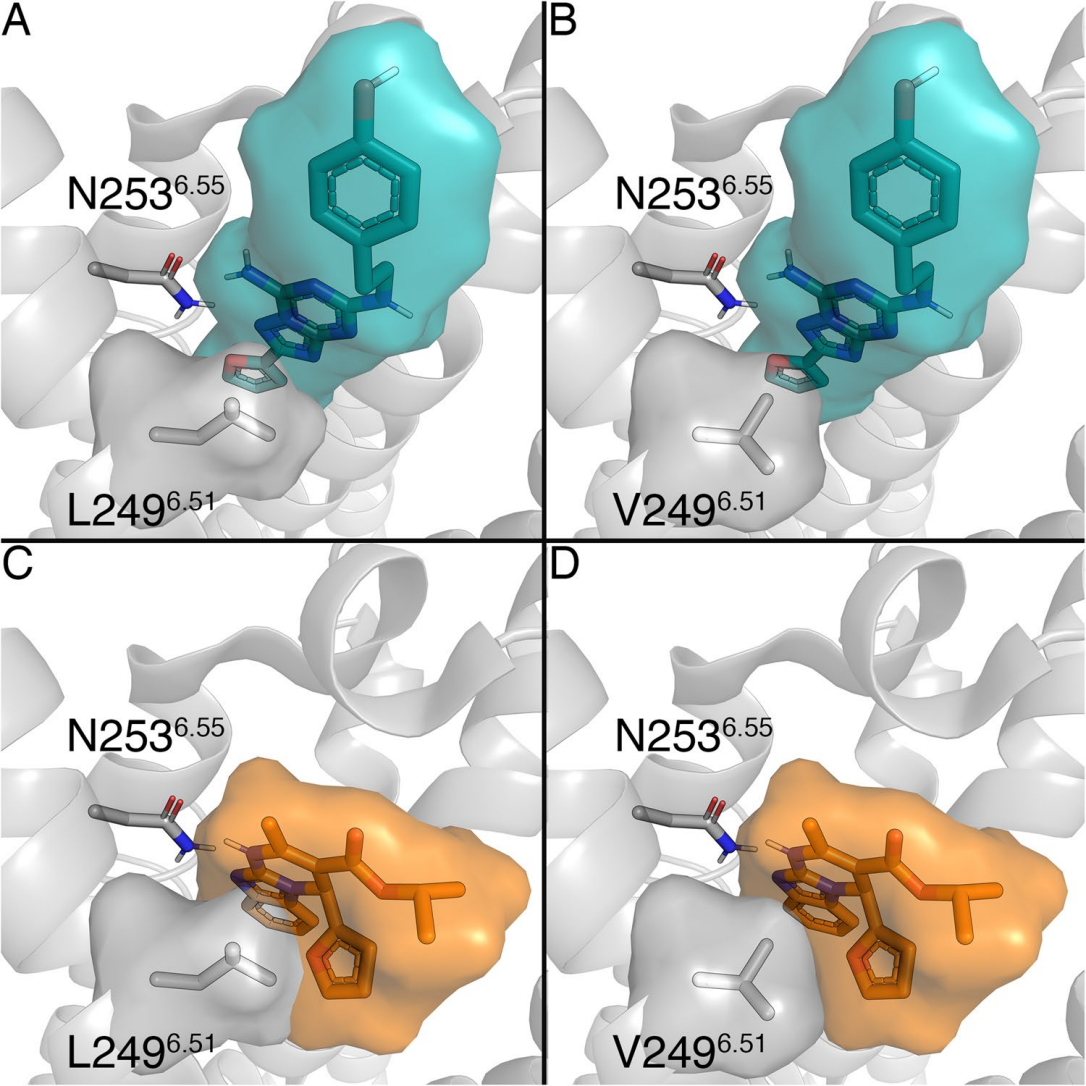
Binding mode of two ligands, ZM241385 (in blue, panels A and B) and (*S*)-ISAM-140 (orange, panels C and D), to the WT (panels A and C) and the L249V^6.51^ mutant (panels B and D) A_2A_AR. Volumetric occupancies are shown as surface. Figure created with Pymol v2.0.

The binding mode of (*S*)-ISAM-140 was obtained by superposition of the previously published complex of this molecule with our A_2B_AR homology-based model^11^ onto a modeled L249V^6.51^ A_2A_AR mutant, i.e. introducing the A_2B_AR sidechain in this position. Such a construct was built and equilibrated on the basis of the high-resolution crystal structure of the ZM241385 — A_2A_AR complex (see “Methods”)^16^. The binding mode obtained included the two key interactions typical of ARs antagonists: (i) hydrogen bond(s) with N253^6.55^ and (ii) π–π stacking with F168^EL2^, both residues completely conserved among ARs^1^. The high-affinity A_2A_AR antagonist ZM241385 showed an optimal shape complementarity with the A_2A_AR WT residue L249^6.51^ (Fig. 2A), whereas the corresponding L249V^6.51^ mutant is expected to minimally disrupt this shape complementarity due to a reduced volume (Fig. 2B). On the other hand, the obtained binding modes for (*S)*-ISAM-140 on the WT A_2A_AR (also obtained assuming the same binding mode as in the A_2B_AR homology-based model^11^) showed a non-optimal fit, in accordance with the lack of affinity exhibited for the A_2A_AR receptor by this derivative and other compounds within the series^8–11^. In particular, the presence of the native L249^6.51^ in the A_2A_AR appeared to introduce a steric clash with either the 2-furyl or 3-thienyl substituents of the ligands, which we hypothesized would reduce binding affinities (Fig. 2C). Conversely, introducing the A_2B_AR sidechain on the modeled L249V^6.51^ A_2A_AR mutant provided a better shape complementarity (Fig. 2D), allowing us to hypothesize that the binding affinity of these antagonists might be recovered to some extent.

### Chiral separation of ISAM-140

**Figure 3:**
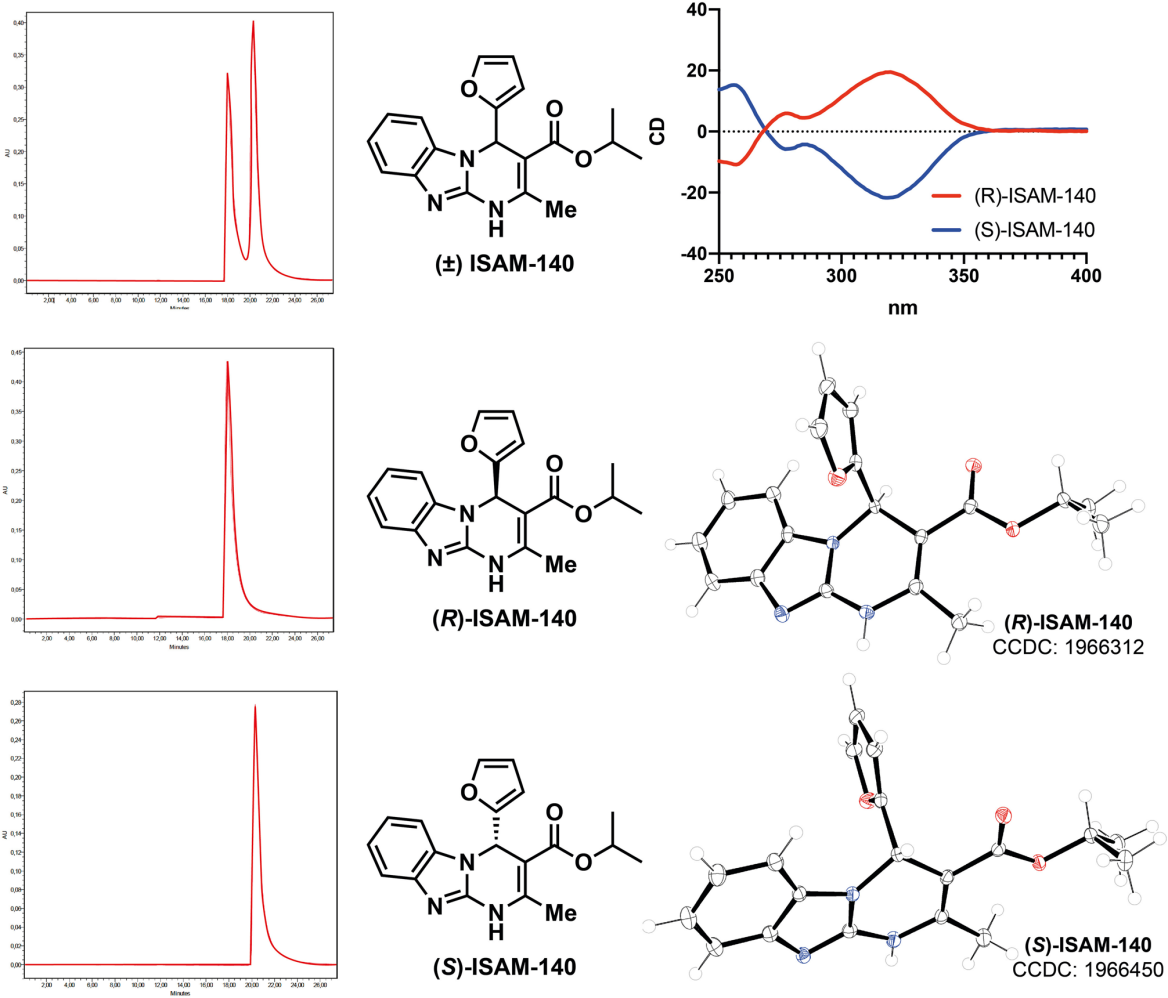
Chiral HPLC separation, circular dichroism spectra and crystal X-ray structure of compounds *(R)*-ISAM-140 and *(S)*-ISAM-140.

The racemic mixture of ISAM-140, obtained as previously described^11^, was resolved into its enantiopure forms. A combination of chiral HPLC, circular dichroism (CD) spectroscopy and X-ray crystallography was employed to separate and unequivocally assign the configuration of the heterocyclic stereocenter in each stereoisomer. Semipreparative HPLC separation of ( ±) ISAM-140 on a chiral stationary phase (see “Experimental information”) provided the expected enantiomers (Fig. 3) with excellent stereochemical purity (> 97%), the analytical and spectroscopy data provided in the Supplementary Information. As described previously for 3,4-dihydropyrimidin-2-ones^17–19^, the characteristic CD activity of the enamide chromophore (300–350 nm) allowed the unambiguous assignment of the absolute configuration of each enantiomer (Fig. 3) by comparison with the reported CD data for enantiopure 3,4-dihydropyrimidin-2(1*H*)-ones of known configuration. In the structures shown in Fig. 3, enantiomers that show a negative Cotton effect (blue line) contain the furan ring pointing backwards, which corresponds to (*S*)-ISAM-140. In contrast, the stereoisomers giving a positive Cotton effect (red line) contain the pentagonal heterocycle pointing forward, which corresponds to (*R*)-ISAM-140. Single crystals suitable for X-ray analysis were grown by slow evaporation of each enantiomer in ethanol. The structures were solved and the data extracted from X-ray crystallography of both monocrystals presented in the Supporting Information (Supplementary Table S1)^13^. The crystal structures of (*S*)-ISAM-140 and (*R*)-ISAM-140 (monoclinic, Fig. 3) confirmed the configuration assignment established by circular dichroism. The benzimidazole moiety is essentially planar in both enantiomers, while the dihydropyrimidine core adopts a pseudo envelope conformation, with the C_4_ atom being lightly displaced by 0.26 Å.

### Determination of ligand binding affinities

**Figure 4:**
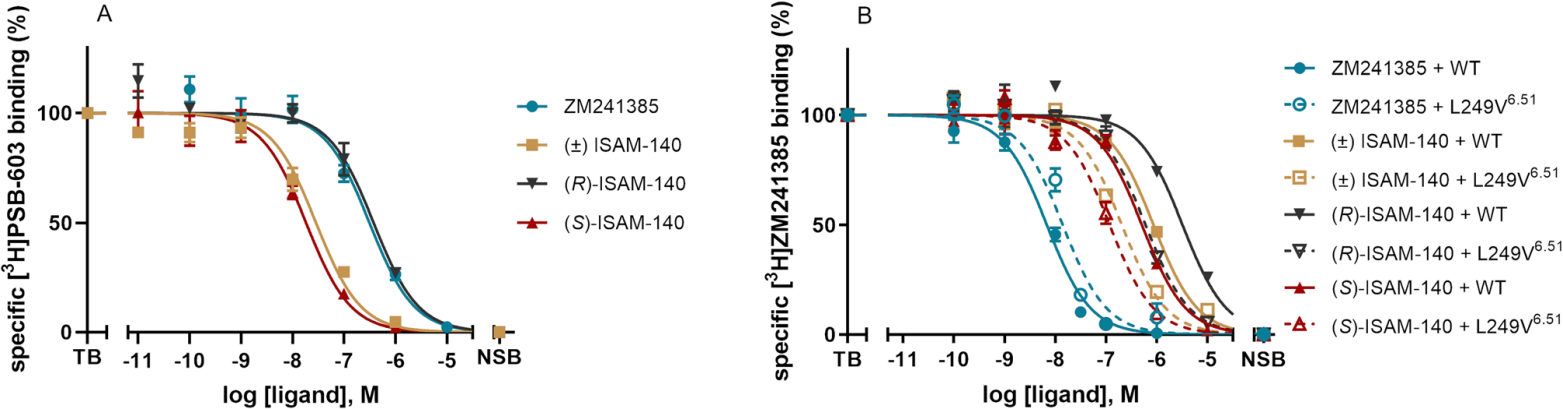
Displacement of (A) specific [^3^H]PSB-603 binding from A_2B_AR and (B) specific [^3^H]ZM241385 binding from the WT and the L249V^6.51^ mutant A_2A_AR at 25 °C by ZM241385 (blue), ( ±) ISAM-140 (yellow), (*R*)-ISAM-140 (black) and (*S*)-ISAM-140 (red). Combined graphs are from three individual experiments performed in duplicate.

To further confirm the role of position 6.51 as a receptor selectivity hotspot, we attempted to express L249V/A^6.51^ A_2A_AR and V250L/A^6.51^ A_2B_AR mutant receptors. Whilst both A_2A_AR mutant receptors were successfully expressed (Supplementary Fig. S1), none of the A_2B_AR mutants could be expressed using standard (non-viral) transfection methods, and consequently the A_2B_AR mutants designed had to be excluded from further experimentation. Thereafter, we determined the binding affinity of ISAM-140, both as a racemate and pure enantiomers, together with the prototypical antagonist ZM241385 at both WT and mutant A_2A_ARs, as well as at the WT A_2B_AR (Fig. 4 and Table 1). The affinities determined for ZM241385 and racemic ISAM-140 on WT A_2B_AR (pKi of 6.78 and 7.86, respectively, see Table 1) were in line with previous reports^10^. As expected from the modeling, the corresponding data for the enantiopure forms of ISAM-140 showed that the affinity of the racemic mixture was due to (*S*)-ISAM-140, with even a gain in binding affinity as compared to the racemic mixture (∆pK_i_ = 0.19), which was dramatically reduced for the low-affinity (*R*)-ISAM-140 (∆pK_i_ = 1.31 between both enantiomers, Fig. 4A and Table 1).

**Table 1:** B_max_ and pK_D_ values of [^3^H]ZM241385 and binding affinities of ZM241385, ( ±) ISAM-140, (*R*)-ISAM-140 and (*S*)-ISAM-140 on WT A_2B_AR, WT and L249V^6.51^ mutant A_2A_ARs.

Receptor	Bmax (pmol/mg)^a^	pK_D_^a^	pK_i_^b^			
	[^3^H]ZM241385		ZM241385	( ±) ISAM-140	(*R*)-ISAM-140	(*S*)-ISAM-140
A_2B_AR (WT)	–	–	6.78 ± 0.06	7.86 ± 0.09	6.74 ± 0.09	8.05 ± 0.06
A_2A_AR (WT)	3.92 ± 0.23	8.59 ± 0.09	8.62 ± 0.04	6.53 ± 0.03	5.96 ± 0.02	6.76 ± 0.04
A_2A_AR (L249V)	1.15 ± 0.15	8.17 ± 0.06	8.09 ± 0.03	6.92 ± 0.03	6.47 ± 0.07	7.17 ± 0.09

Data is presented as mean ± SEM of three individual experiments, each performed in duplicate. pK_D_ values obtained from homologous competition displacement assays on transiently transfected HEK293-hA_2A_AR membranes at 25 °C. pK_i_ values obtained from displacement assays of specific [^3^H]PSB-603 binding from CHO-spap-hA_2B_AR membrane or specific [^3^H]ZM241385 binding from transiently transfected WT and mutant HEK293-hA_2A_AR membranes at 25 °C.

For the A_2A_AR, we first established whether the L249V/A^6.51^ mutants still sufficiently bound ZM241385, to validate the viability of using it as a radioligand in the homologous displacement assays. Of note, the resulting K_D_ values could then be used to obtain K_i_ values from the IC_50_ values (see Methods), which enabled us to compare affinity values for WT and mutant A_2A_ARs. Moreover, the resulting B_max_ values showed that the A_2A_AR L249V^6.51^ mutant had a lower expression level than the WT A_2A_AR. A slight reduction in affinity of both [^3^H]ZM241385 and ZM241385 was observed on this mutant (Table 1), which was in line with our hypothesis that the shape complementarity between ZM241385 and L249 is mostly preserved with a smaller Val. However, a substantial hydrophobic side chain was important for the binding of this antagonist to the A_2A_Rs, since its affinity to the A_2A_AR L249A^6.51^ mutant was completely lost (Supplementary Fig. S2), in line with previous reports^20^. The results of the displacement assays for ISAM-140 (racemate and both stereoisomers) are illustrated in Fig. 4B and Table 1. Although one data point for ( ±) ISAM-140 at the concentration of 10^−5^ M was excluded from the curve of WT A_2A_AR, due to low water solubility, in all cases the binding affinity for the WT A_2A_AR was very low (within micromolar range). Notably, it followed the same trend as observed on WT A_2B_AR, i.e. the highest affinity for (*S*)-ISAM-140 and the lowest for (*R*)-ISAM-140. The selectivity ratio between A_2B_ and A_2A_ ARs was substantial for ( ±) ISAM-140, (∆pK_i_ = 1.33), in line with the previous reports for this ligand^11^. This difference was maintained for the eutomer (*S*)-ISAM-140 (∆pK_i_ = 1.29) and, to a lower extent, even for (*R*)-ISAM-140 (∆pK_i_ = 0.79), which is expected due to its already low affinity for A_2B_AR. Notably, the affinity values were significantly recovered at the A_2A_AR L249V^6.51^ mutant, i.e. when the receptor was more “A_2B_AR-like”, thus supporting the initial modeling hypothesis. The moderate affinity gains observed for the A_2A_AR L249V^6.51^ mutant as compared to the A_2A_AR WT [0.39, 0.41 and 0.51 log unit for ( ±) ISAM-140, (*S*)-ISAM-140, and (*R*)-ISAM-140, respectively, see Table 1] did not restore the affinity values as in the WT A_2B_AR.

### Computational characterization of binding free energies

**Figure 5:**
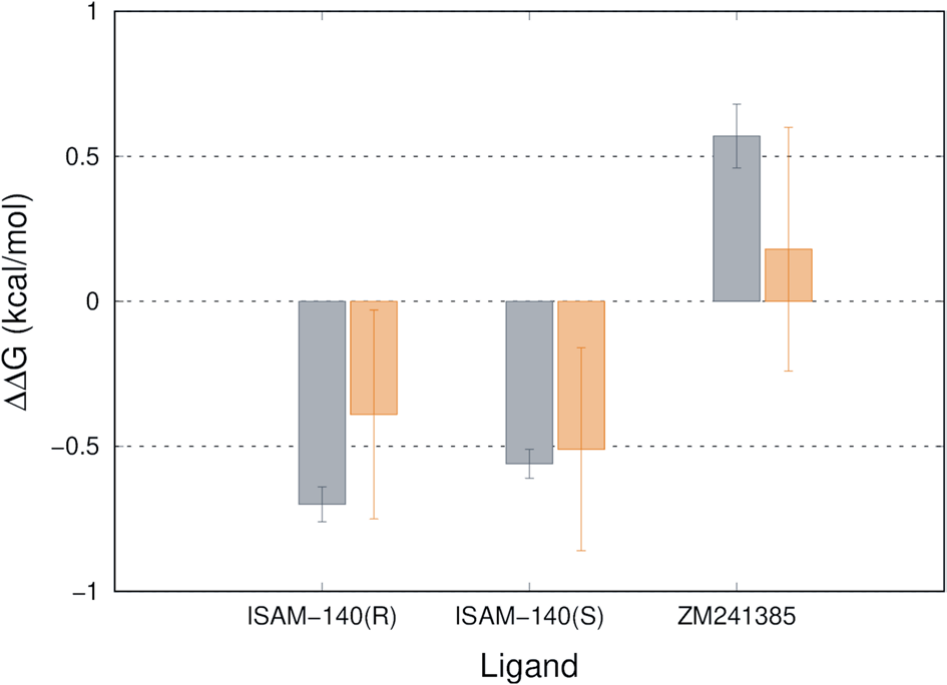
Experimental (grey) and calculated (orange) relative changes in binding free energies to the L249V^6.51^ mutant A_2A_AR for the two enantiomers of ISAM-140 and ZM241385.

Finally, we investigated the observed shifts in binding affinities for (*S*)-ISAM-140, (*R*)-ISAM-140 and ZM241385 in the context of the structural binding model of these molecules to the A_2A_AR. The approach was to compare the WT and L249^6.51^V mutant (A_2B_ equivalent) versions of A_2A_AR using the Q-FEP protocols^21,22^. This strategy consists on the simulation of the mutation (Leu to Val) both in the presence and absence of each of the docked ligands. While the structure of the ZM241385 — A_2A_AR complex is experimentally known^16^, the binding mode of each enantiomer of ISAM140 was inferred from our previous work on this chemotype^7^. Figure 5 summarizes the calculated shift in the free energy of binding due to the L249V^6.51^ mutation for each enantiomer of ISAM-140 and for ZM241385. It can be observed a very good agreement between the calculations and the experimental affinity data here reported in Fig. 4B, with a very low mean average error (MAE = 0.25 kcal/mol, numerical data provided in Supplementary Table S2). Thus, the simulation of this mutation resulted in a predicted increase in affinity (negative ∆∆G_bind (mut − WT)_ values in Fig. 5) for both enantiomers of ISAM-140, with values proportional to those extracted from the experimental data. Conversely, the experimental affinity of ZM241385 is decreased for the L249V^6.51^ mutant A_2A_AR, which is also captured by our modeling as a mild positive value for the calculated ∆∆G_bind (mut − WT)_.

## Discussion

In this work, we investigated the role of position 6.51 in determining the specificity for A_2B_AR binding of a series of chiral antagonists recently developed for this receptor. The modeling hypothesis behind the design of the potent antagonist ISAM-140 placed the *S*-stereoisomer in perfect shape complementarity with Val250^6.51^ in the A_2B_AR, while analogous docking in the high resolution A_2A_AR bearing a bulkier Leu in the same position showed initial steric clashes. This allowed us to propose this sidechain as a landmark for A_2B_AR selectivity for this ligand class, and the (*S*)-ISAM-140 as the active stereoisomer. To experimentally validate this hypothesis, the ISAM-140 enantiomers were separated and their absolute configuration unequivocally assigned. Besides this goal, the enantiomeric separation and pharmacological characterization of this reference A_2B_AR antagonist allowed to confirm the expected higher affinity of the *S* enantiomer, in line with the original modeling hypothesis^11^ and recent similar results obtained with derivatives of this scaffold^10,13,14^.

Site-directed mutagenesis of position 6.51 was performed on the A_2A_AR to replace the WT Leu by the Val specific of A_2B_AR, as the reverse mutation of the A_2B_AR appeared unfeasible in our hands, somehow in contrast to previous report of Müller and co-workers who managed to express the corresponding Ala mutant (V250A^6.51^) in the A_2B_AR^23^. It is worth noting that, while there had been reports of the Alanine scan of position 6.51 in both A_2A_^20^ and A_2B_ARs^23^, this is the first time that the introduction of the A_2B_AR characteristic Val sidechain on the A_2A_AR is evaluated.

The L249V^6.51^ A_2A_AR mutant partially recovered the affinity of ISAM-140 lost for this receptor, supporting the initial modeling hypothesis. This partial recovery in affinity, consistently observed for all three forms of this molecule (i.e., racemic mixture and both eutomers) is in line with recent reports on ‘selectivity hotspots’ between A_1_AR and A_2A_AR, where a single-point mutation clearly affecting the experimental binding mode could only partially explain the observed selectivity profile of the A_1_AR selective xanthines under investigation^24^. On the other hand, the opposed effect was observed for ZM241385 (i.e. decrease in affinity for the L249V^6.51^ A_2A_AR mutant) in line with the well-described preference of this ligand for the A_2A_AR.

**Figure 6:**
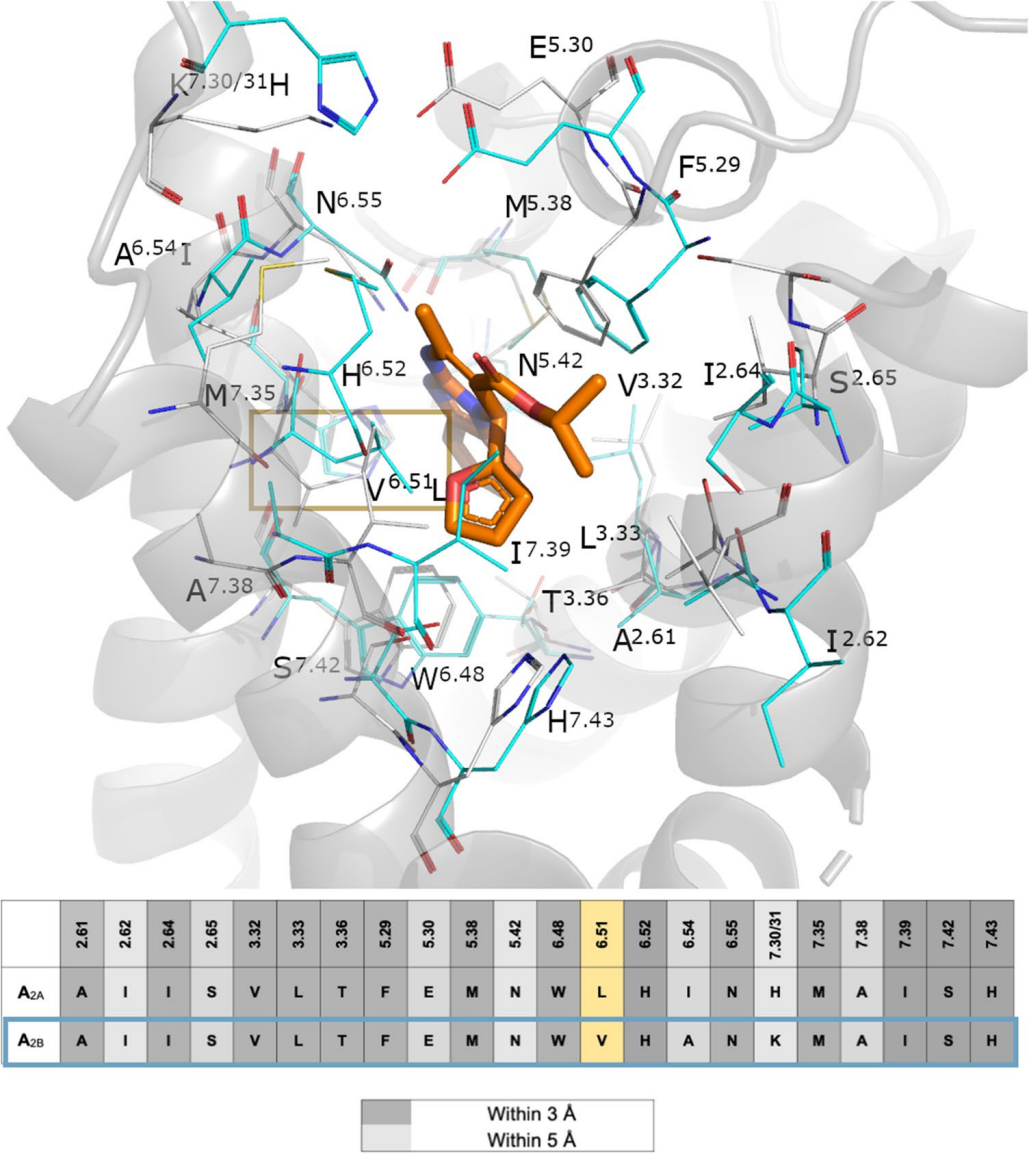
Pseudo-sequence alignment of the residues within 5 Å of any atom of *(S)*-ISAM140, as predicted by docking on the A_2B_AR, between this receptor and the A_2A_AR. The location of each sidechain is shown in the 3D superposition of the *(S)*-ISAM140-A_2B_AR (gray sidechains and cartoon, ligand in orange sticks) with the A_2A_AR crystal structure (cyan sidechains). Position 6.51 is highlighted on a yellow box. Figure created with Pymol v2.0.

To further assess the amino acid conservation between the A_2A_ and A_2B_ARs binding sites, a pseudo-sequence alignment is presented in Fig. 6. One can observe that, in addition to position 6.51 here studied, only two sidechains vary within the 5 Å cut-off distance with the ligand: Ala253^6.54^ in A_2B_AR, situated one helix turn below position 6.51, is an Ile in A_2A_AR. This residue, however, is not in contact with the ligand and instead involved in the TM packing as shown in the Fig. 6. In the EL3 region, His264^7.31^ in A_2A_AR is making a salt bridge interaction with Glu169^5.30^ in EL2, a role that in our A_2B_AR model is undertaken by Lys267^7.31^ (Fig. 6). While this residue has been shown to be involved in ligand binding kinetics^1^, we should not rule out an additional role of the more variable EL regions in the selectivity profile of this antagonist. This analysis also allows to explore potential indirect effects of the V^6.51^L mutation on neighbouring residues conserved in the ARs, like His^6.52^ that has been shown to be involved in both agonist and antagonist binding^1^. As it can be seen in Fig. 6., this residue is not predicted to change conformation between A_2A_ and A_2B _ARs, which is supported by the water-mediated interaction with Asn^5.42^ previously characterized by MD simulations of this pair of receptors^25^.

In the lack of a crystal structure of the A_2B_AR, the observed effects were rationalized back in the modeled structures, by means of first-principle FEP simulations of this mutation. The QresFEP protocol has been broadly applied to investigate the A_2A_AR mutational landscape^26–28^, showing exceptional sensitivity to capture the correct affinity shifts for different chemotypes. The binding model of *(S)*-ISAM-140 to the WT and L249V^6.51^ mutant versions of A_2A_AR was here assumed to be the same as our docking model of this compound to the WT A_2B_AR^13^. That model suggested that the high A_2B_AR affinity of ( ±) ISAM-140 was due to the stereoselective optimal fitting of the (*S*) isomer to the A_2B_AR binding site, facilitated by the Val sidechain in position 6.51 of this receptor^13^. The calculated recovery of the binding affinity of *(S)*-ISAM-140 upon the L249V^6.51^ mutation in the A_2A_AR, which is in line with the experimental design of this A_2B_-like mutation on the A_2A_AR, further confirms the validity of the binding model for this chemotype on the A_2B_AR.

Overall, both experimental and computational results of this study clearly support the binding mode used to design this study, providing useful structural insights in the selective recognition of these A_2B_AR antagonists that should aid in future structure-based optimization.

## Methods

### HPLC separation and characterization of ISAM-140 enantiomers

The chiral resolution of the razemic ligand ISAM-140 was performed following procedures recently described^13^. Briefly, chiral resolution was performed using a Water Breeze 2 (binary pump 1525, detector UV/Visible 2489, 7725i Manual Injector Kit 1500 Series). Compound ISAM-140 enantiomers were separated using a 250 mm × 20 mm Chiralpak 5 µm IE-3 (DAICEL). All the separations were performed at 25 ºC with hexane/isopropanol 7:3 as mobile phase. The enantiomers [(*R*)-ISAM-140 (3 mg, t_R_ = 17.90 min), (*S*)-ISAM-140 (3.1 mg, t_R_ = 20.31 min)] were isolated, their stereochemical purity analyzed by chiral HPLC (ee: 97–99% for each enantiomer) and then characterized by NMR (see Supplementary Material).

### Circular dichroism

Circular dichroism spectra were recorded on a Jasco-815 system equipped with a Peltier-type thermostatic accessory (CDF-426S, Jasco). Measurements were carried out at 20 °C using a 1 mm quartz cell in a volume of 600 µL. Compounds (0.5 mg) were dissolved in MeOH (1.0 mL) and then diluted 10-fld in MeOH. The instrument settings were bandwidth, 1.0 nm; data pitch, 1.0 nm; speed, 500 nm/min; accumulation, 10; wavelengths, 400–190 nm.

### X-ray crystallography of ISAM-140 enantiomers

Crystals of (*S*)-ISAM-140 and (*R*)-ISAM-140 were grown by slow evaporation from ethanol solutions. For the crystal structure determination^13^, the data were collected by applying the omega and phi scans method on a Bruker D8 VENTURE PHOTON III-14 diffractometer using Incoatec multilayer mirror monochromated with Cu-Kα radiation (λ = 1.54178 Å) from a microfocus sealed tube source at 100 K with detector resolution of 7.3910 pixels mm-1. Computing data and reduction were made with the APEX3 v2018.7–2 (BRUKER AXS, 2005). The structure was solved using SHELXT2018/22 and finally refined by full-matrix least-squares based on F2 by SHELXL2018/3.3 An empirical absorption correction was applied using the SADABS2016/2 program. Software used for molecular graphics: ORTEP for Windows. Software used to prepare material for publication: WinGX2018.3 publication routines4 and Mercury.

The obtained structures were refined following recently described methods as follows^13^: All non-hydrogen atoms were refined anisotropically and the hydrogen atom positions were included in the model on the basis of Fourier difference electron density maps. All aromatic CH hydrogen (C-H = 0.95 Å), methine hydrogen (C-H = 1.0 Å) and methylene hydrogen (C-H = 0.99 Å) atoms were refined using a riding model with Uiso(H) = 1.2 Ueq(C). The methyl hydrogen (C–H = 0.98 Å) atoms were refined as a rigid group with torsional freedom [Uiso(H) = 1.5 Ueq(C)] and the hydrogens atom of NH groups (HiN) as a free atom with Uiso(H) = 1.2 Ueq(C).

### Site-directed mutagenesis

Site-directed mutants of the A_2A_AR were generated by polymerase chain reaction (PCR) mutagenesis as described previously^29^. pcDNA3.1(+)-hA_2A_AR with N-terminal HA and FLAG tags and a C-terminal His tag was used as the template. Primers for mutants L249V^6.51^ and L249A^6.51^ were designed by the QuikChange Primer Design Program of Agilent Technologies (Santa Clara, CA, USA) and primers were obtained from Eurogentec (Maastricht, The Netherlands). All DNA sequences were verified by Sanger sequencing at LGTC (Leiden, The Netherlands).

### Cell culture and transient transfection

CHO cells stably expressing the human A_2B_AR (CHO-spap-hA_2B_AR) were cultured in Dulbecco’s modified Eagle’s medium: Nutrient Mixture F-12 (DMEM/F12) supplemented with 10% newborn calf serum, 50 µg/mL streptomycin, and 50 IU/mL penicillin at 37 °C and 5% CO_2_ atmosphere. Cells were subcultured twice a week at a confluency of 80–90%. For transient transfections, human embryonic kidney (HEK) 293 cells were cultured as monolayers in DMEM supplemented with stable glutamine, 10% newborn calf serum, 50 µg/mL streptomycin, and 50 IU/mL penicillin at 37 °C and 7% CO_2_ atmosphere as reported previously^29,30^. The cells were seeded on 10 cm ø plates and transfected with 10 μg plasmid DNA of wild-type (WT) or mutant hA_2A_AR using the calcium phosphate precipitation method^31^, followed by a 48 h incubation.

### Membrane preparation

HEK293 cells transiently expressing WT or mutant human A_2A_AR (HEK293-hA_2A_AR) were detached from the plates 48 h post-transfection by scraping into phosphate-buffered saline (PBS) and collected by centrifugation at 1,000 × g for 5 min. The pellets from 10 plates were pooled and resuspended in ice-cold Tris–HCl buffer (50 mM, pH 7.4) and then homogenized with an UltraTurrax homogenizer (Heidolph Instruments, Schwabach, Germany). The cell membrane suspensions were centrifuged at 100,000×*g* at 4 °C for 20 min in a Beckman Optima LE-80 K ultracentrifuge. The pellet was resuspended in ice-cold Tris–HCl buffer, and the homogenization and centrifugation steps were repeated one more time. After this, Tris–HCl buffer was used to resuspend the pellet of HEK293 cell membranes. Membrane preparation for CHO-spap-hA_2B_AR cells followed a similar procedure after they were grown to 90% confluence in 15 cm plates, and membranes pellets were finally resuspended in Tris–HCL buffer containing 10% (w/v) CHAPS. In both cases, 0.8 IU/ml adenosine deaminase was added to break down endogenous adenosine and membranes were aliquoted into 250 μL and stored at -80 °C until further use. Membrane protein concentrations were determined using the BCA method^32^.

### Radioligand binding assays

Radioligand binding experiments on CHO-spap-hA_2B_AR membranes were adjusted from previously reported data^33^. Membrane aliquots containing 30 µg of protein were incubated in a total volume of 100 µL of assay buffer. Nonspecific binding was determined with 10 µM ZM241385. Then 25 µL cell membrane suspension, 25 µL of 1.5 nM radioligand [^3^H]PSB-603, 25 µL of assay buffer [50 mM Tris–HCl, 0.1% (w/v) CHAPS, pH 7.4 at 25 °C] and 25 µL of the indicated compounds in increasing concentrations in the same assay buffer were added to each well and followed by a 120 min incubation at 25 °C. Radioligand displacement experiments with transient HEK293-hA_2A_AR cell membranes were performed as described previously^34^. Briefly, membrane aliquots containing 5–7.5 µg of protein were incubated in a total volume of 100 µL of assay buffer to adjust the assay window to approximately 2000 DPM. Nonspecific binding was determined in presence of 100 µM NECA and represented less than 10% of the total binding. Then 25 µL membrane suspension (5–7.5 µg of protein), 25 µL of 5.0 nM radioligand [^3^H]ZM241385, 25 µL of assay buffer [50 mM Tris–HCl, pH 7.4] and 25 µL of the indicated compounds at different concentrations in the same assay buffer were added to each well, with final assay concentration of radioligand of 5 nM. For homologous displacement experiments, radioligand displacement experiments were performed in presence of three concentrations of [^3^H]ZM241385 (1.7 nM, 5.0 nM and 9.5 nM) and increasing concentrations of unlabeled ZM241385. After 120 min at 25 °C, incubations were terminated by rapid vacuum filtration through GF/B filter plates (PerkinElmer, Groningen, Netherlands) using a Perkin Elmer Filtermate-harvester. Filterplates were subsequently washed ten times with ice-cold assay buffer. Filter-bound radioactivity was determined by scintillation spectrometry using a Microbeta^2^ 2450 microplate counter (PerkinElmer).

### Data analysis

Data analyses were performed using GraphPad Prism 7.0 software (GraphPad Software Inc., San Diego, CA). pK_D_ values and B_max_ were obtained by non-linear regression analysis using “one-site homologous” model. pIC_50_ values were determined by fitting the data using non-linear regression to a sigmoidal concentration–response curve equation. pK_i_ values were calculated from pIC_50_ values using the Cheng-Prusoff equations^35^.

### Computational modeling

The high resolution crystal structure of A_2A_AR (PDB code 4EIY^16^) was used as a starting point for the calculations. The protein was prepared for MD simulations as follows: (i) removing co-factors and fused proteins employed for crystallization, (ii) reverting the crystal construct to the wild-type (WT) A_2A_AR receptor, (iii) the assignment of protonation states of ionizable residues, (iv) mutation of the WT Leu249^6.51^ to Val as in the corresponding A_2B_AR and (v) membrane insertion using PyMemDyn^25^. The latter stage involves embedding of the protein in a pre-equilibrated POPC membrane, soaking of the system with bulk water and a short (5 ns) equilibration period with GROMACS 4.6^36^ using the OPLS-AA force field^37^ and Berger parameters for the lipids^38^. Thereafter, ligands were manually docked to the equilibrated receptor using as a reference the putative binding mode of SYAF014^7^ to the A_2B_AR previously described. In the case of ZM241385, the coordinates of the crystal structure ligand were retained during the equilibration process. Subsequently, each equilibrated L249V^6.51^ A_2A_AR-ligand complex was transferred to the MD software Q^39^ for free energy perturbation (FEP) calculations under spherical boundary conditions using QligFEP^21^. A 25 Å radius sphere centered on the center of geometry of the ligand was constructed for these MD simulations. Solvent atoms were subject to polarization and radial restraints using the surface-constrained all-atom solvent (SCAAS)^40^ model to mimic the properties of bulk water at the sphere surface. Atoms lying outside the simulation sphere were tightly constrained (200 kcal/mol/Å^2^ force constant) and excluded from the calculation of non-bonded interactions. Long range electrostatic interactions beyond a 10 Å cut off were treated with the local reaction field method^41^, except for the atoms undergoing the FEP transformation, where no cutoff was applied. Solvent bond and angles were constrained using the SHAKE algorithm^42^. All titratable residues outside the sphere were neutralized as reported elsewhere^21^. Residue parameters were translated from the OPLS-AA/M force field^43^ and the parameters for the lipids were inherited from the previous MD stage, while ligand parameters were generated using the ffld_server as implemented in the Schrödinger suite. The simulation sphere was warmed up from 0.1 to 298 K, during a first equilibration period of 0.61 ns, where an initial restraint of 25 kcal/mol/Å^2^ imposed on all heavy atoms was slowly released for all complexes. Thereafter the system was subject to 10 parallel replica MD simulations, in which the FEP protocol was applied for each residue transformation. Each of these MD replicates started with a 0.25 ns unbiased equilibration period, with different initial velocities. The FEP protocol for the L → V mutation was generated by combing the QresFEP^22^ protocol for residue mutations with a dual topology approach inspired from QligFEP^21^, where the effective topology along the transformation is a linear combination of the two original sidechain topologies. Each FEP transformation consisted of 51 evenly distributed λ-windows with 10 ps MD sampling each. In order to fulfill a thermodynamic cycle and calculate relative binding free energies, parallel FEP transformations were run for the apo-structure, i.e. the protein structure without ligand. In these simulations the same parameters were applied (i.e., sphere size, simulation time, etc.), and a total of 10 replicates × 2 (apo/holo) states × 2 (WT and mut) annihilations × 51 λ-windows × 10 ps = 20.4 ns sampling was performed for each mutation simulation. The relative binding free energy shift between WT and mutant receptors for each ligand was estimated by solving the thermodynamic cycle utilizing the Bennett acceptance ratio (BAR)^44^. All 3D images were produced in PyMOL^45^.

## Supplementary Information

Supplementary Information.
