# Supplementary material for: Identification of V6.51L as a selectivity hotspot in stereoselective A2B adenosine receptor antagonist recognition
Source: Sci Rep. 2021 Jul 8;11:14171. doi: 10.1038/s41598-021-93419-x (PMC8266863; doi:10.1038/s41598-021-93419-x)
Supplement: Supplementary file 1 — Supplementary Information. [file 41598_2021_93419_MOESM1_ESM.pdf]

SUPPORTING INFORMATION

Identification of V6.51L as a selectivity hotspot in stereoselective A_2 receptor antagonist recognition

Xuesong Wang[1*], Willem Jespers[1,2*], Ruben Prieto-Díaz[3,4*], Maria Majellaro[3,4], Adriaan P. IJzerman[1], Gerard J. P. van Westen[1], Eddy Sotelo[3,4#], Laura H. Heitman[2,5#] and Hugo Gutiérrez-de-Terán[2#]

Table of Contents

Supplementary Figure S1 ········· 2
Supplementary Figure S2 ········· 2
Supplementary Table S1 ········· 3
Supplementary Table S2 ········· 4
Spectroscopic and analytical data ········· 4
Enzyme-linked Immunosorbent assay (ELISA). ········· 5
References ········· 6

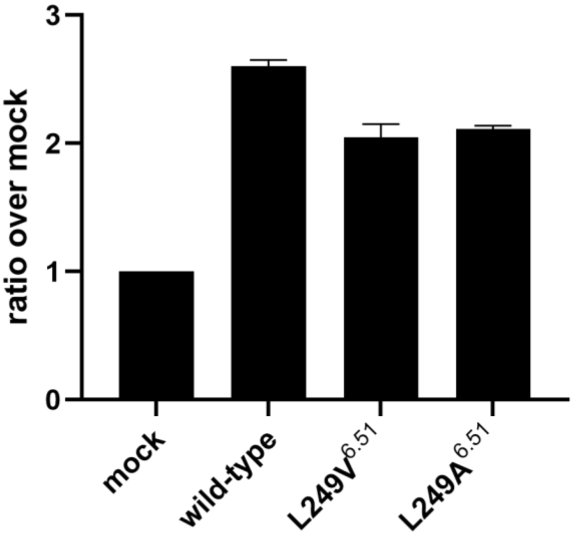

Supplementary Figure S1. Expression level of the transiently transfected WT A_{2A}AR, and L249V$^{6.51}$ and L249A$^{6.51}$ mutant A_{2A}AR at the surface of HEK293 cells. Data are shown as the mean ± SEM of three individual experiments performed in sextuplicate.

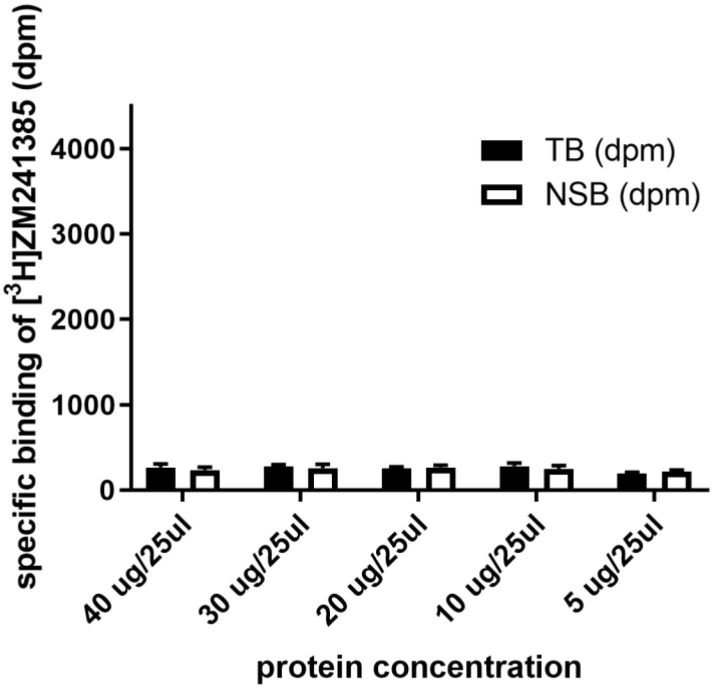

Supplementary Figure S2. Window check of HEK293 cell membrane transiently transfected by the L249$^{6.51}$A mutant A_{2A}AR in the presence of 1.7 nM [^{3}H]ZM241385 in the absence (total binding; TB) and presence (non-specific binding; NSB) of NECA (100 µM). Data is shown as the mean ± SEM of three individual experiments performed in duplicate.

Supplementary Table S1. X-ray diffractometry experimental details of crystallographic (*R*)-ISAM-140 and (*S*)-ISAM-140.

**Crystal data**	**(*R*)-ISAM140**	**(*S*)-ISAM140**
CCDC	1966312	1966450
Chemical formula	$C_{19}H_{19}N_3O_3$	$C_{19}H_{19}N_3O_3$
M_r	337.37	337.37
Crystal system	Monoclinic	Monoclinic
Space group	C2	C2
Temperature (K)	100	100
a (Å)	16,4552 (9)	16.4553 (4)
B (Å)	8.0613 (4)	8.0605 (2)
c (Å)	13.5259 (7)	13.5260 (3)
α (°)	90	90
β (°)	112.684 (3)	112.678 (1)
γ (°)	90	90
V ($Å^3$)	1655.42 (16)	1655.35 (7)
Z	4	4
Radiation type	Cu-Kα	Cu-Kα
μ (mm^{-1})	0.76	0.76
Crystal size (mm)	0.12 × 0.11 × 0.10	0.11 × 0.01 × 0.03
Tmin, Tmax	0.852, 0.929	-
(sin θ/λ) max ($Å^{-1}$)	0.633	0.625
Measured/Independent/ observed [I>2σ(I)] reflection	20568/3488/3266	17353/3370/3346
Rint	0.068	0.076
$R[F^2>2\sigma(F^2)],wR(F2), S$	0.043, 0.102, 1.10	0.025, 0.069, 1.01
$\Delta\rho$max/$\Delta\rho$min ($eÅ^{-3}$)	0.19, −0.25	0.16, -0.20
Absolute structure (Flack)	-0.1(2)	-0.02 (4)

Supplementary Table S2. Experimental and FEP calculated energies for the L6.51V mutation, with the value for each FEP leg in the thermodynamic cycle included. The ΔΔG values are plotted on Fig 5 on the main text. All (Δ)Δ G values and expressed in Kcal·mol^{-1}. Standard error of the mean (sem) calculated from 10 replica simulations (FEP) or from the experimental data (see main text). $\Delta\Delta G_{exp}$ = -RTln(K_i^{wt}/K_i^{mut})

		$\Delta G_{calc} = \Delta G_{apo} - \Delta G_{holo}$		
	$\Delta\Delta G_{exp}$	$\Delta\Delta G_{calc}$	ΔG_{holo}	ΔG_{apo}
ISAM-140(R)	-0.70±0.06	-0.39±0.36	-4.52±0.24	-4.91±0.27
ISAM-140(S)	-0.56±0.05	-0.50±0.35	-4.41±0.22	-4.91±0.28
ZM241385	0.57±0.11	0.20±0.42	-5.11±0.31	-4.91±0.29

Spectroscopic and analytical data for racemates and enantiomers isolated through chiral HPLC.

(±) Isopropyl 4-(furan-2-yl)-2-methyl-1,4-dihydrobenzo[4,5]imidazo[1,2-*a*]pyrimidine-3-carboxylate [(±) ISAM-140].[1] ^{1}H NMR (300 MHz, DMSO-*d6*), δ (ppm): 10.78 (brs, 1H), 7.67−7.23 (m, 3H), 7.19−6.84 (m, 2H), 6.52 (s, 1H), 6.44 (d, *J* = 3.3 Hz, 1H), 6.37−6.23 (m, 1H), 4.86 (h, *J* = 6.3 Hz, 1H), 2.44 (s, 3H), 1.21 (d, *J* = 6.2 Hz, 3H), 1.05 (d, *J* = 6.1 Hz, 3H). ^{13}C NMR (75 MHz, DMSO-*d6*), δ (ppm): 165.0, 153.3, 148.0, 146.0, 143.0, 142.6, 132.0, 122.3, 120.7, 117.2, 110.8, 110.2, 108.2, 94.9, 67.0, 49.7, 22.3, 22.0, 19.1. HRMS (ESI) m/z: calcd for $C_{19}H_{20}N_3O_3$ [M + H]$^+$: 338.1488; found: 338.7927.

Isopropyl (*R*)-4-(furan-2-yl)-2-methyl-1,4-dihydrobenzo[4,5]imidazo[1,2-*a*]pyrimidine-3-carboxylate [(*R*)-ISAM-140]. ^{1}H NMR (300 MHz, DMSO-d_6), δ (ppm): 10.76 (s, 1H), 7.41 (d, J = 11.0 Hz, 2H), 7.34 (d, J = 7.7 Hz, 1H), 7.04 (dt, J = 18.2, 7.0 Hz, 2H), 6.52 (s, 1H), 6.44 (d, J = 3.3 Hz, 1H), 6.35 – 6.27 (m, 1H), 4.87 (p, J = 6.3 Hz, 1H), 2.44 (s, 3H), 1.21 (d, J = 6.3 Hz, 3H), 1.05 (d, J = 6.4 Hz, 3H). ^{13}C NMR (75 MHz, DMSO-d_6), δ (ppm): 165.0, 153.3, 148.0, 146.0, 143.1, 142.6, 132.0, 122.3, 121.0, 117.2, 110.8, 110.2, 108.7, 95.0, 67.0, 49.7, 22.3, 22.1, 19.1. HRMS (APCI) *m/z* calcd for $C_{19}H_{19}N_3O_3$ $[M+H]^+$: 338.1499; found: 338.1501.

Isopropyl (*R*)-4-(furan-2-yl)-2-methyl-1,4-dihydrobenzo[4,5]imidazo[1,2-*a*]pyrimidine-3-carboxylate [(*S*)-ISAM-140]. ^{1}H NMR (300 MHz, DMSO-d_6), δ (ppm): 10.78 (s, 1H), 7.42 (d, J = 10.9 Hz, 2H), 7.35 (d, J = 7.8 Hz, 1H), 7.04 (dt, J = 18.2, 7.0 Hz, 2H), 6.54 (s, 1H), 6.44 (d, J = 3.3 Hz, 1H), 6.40 – 6.29 (m, 1H), 4.87 (p, J = 6.3 Hz, 1H), 2.44 (s, 3H), 1.21 (d, J = 6.3 Hz, 3H), 1.05 (d, J = 6.4 Hz, 3H). ^{13}C NMR (75 MHz, DMSO-d_6), δ (ppm): 165.0, 153.3, 148.0, 146.0, 143.0, 142.6, 132.0, 122.3, 120.7, 117.5, 110.8, 110.3, 108.4, 94.9, 67.0, 49.7, 22.3, 22.2, 19.0. HRMS (APCI) *m/z* calcd for $C_{19}H_{19}N_3O_3$ $[M+H]^+$: 338.1499; found: 338.1501.

Enzyme-linked Immunosorbent assay (ELISA). The experiment was performed as described previously.[2] Briefly, 24 hours after transfection, cells were split into a 96-well poly-D- lysine-coated plates at a density of 10^6 cells per well. After an additional 24 h, the cells were fixed with 4% formaldehyde and blocked with 2% bovine serum albumin (BSA) (Sigma-Aldrich Chemie N.V., Zwijndrecht, The Netherlands) in Tris-buffered saline (TBS). Then, the cells

were incubated with monoclonal M1-anti-FLAG antibody (1:2250) (Sigma-Aldrich Chemie N.V. Zwijndrecht, The Netherlands) in Tris-buffered saline (TBS)/1 mM $CaCl_2$ for 2 hours at room temperature (RT). Next, the antibody was removed and the cells were washed with TBS/1 mM $CaCl_2$ before adding the secondary antibody, monoclonal anti-Mouse-HRP (1:5000) (Jackson ImmunoResearch Europe Ltd., Cambridgeshire, UK) and incubating for 1 hour at RT. After removing the secondary antibody and washing the cells with TBS/1 mM $CaCl_2$, 3, 3',5,5'-tetramethyl-benzidine (TMB) was added and incubated for 5 minutes in the dark. The reaction was stopped with 1 M H_3PO_4, and absorbance was read at 450 nm using a Wallac EnVision 2104 Multilabel reader (PerkinElmer).

References

1. El Maatougui, A. *et al.* Discovery of potent and highly selective A2B adenosine receptor antagonist chemotypes. *J. Med. Chem.* **59**, 1967–1983 (2016).

2. Lane, J. R. *et al.* A novel nonribose agonist, LUF5834, engages residues that are distinct from those of adenosine-like ligands to activate the adenosine A2a receptor. *Mol. Pharmacol.* **81**, 475–487 (2012).
